# ﻿Evidence of late root formation of molars in Anderson’s red-backed vole, *Eothenomysandersoni* (Thomas, 1905) (Cricetidae, Rodentia), and arguments for its generic allocation

**DOI:** 10.3897/zookeys.1123.86960

**Published:** 2022-10-07

**Authors:** Masahiro A. Iwasa, Yukibumi Kaneko, Yoshiyuki Kimura

**Affiliations:** 1 College of Bioresource Sciences, Nihon University, Fujisawa, Kanagawa 252-8510, Japan Nihon University Fujisawa Japan; 2 Takayacho 502-4, Sakaide, Kagawa 762-0017, Japan Unaffiliated Sakaide Japan; 3 Mitouchi 6-10, Houkida, Fukushima, Fukushima 960-8163, Japan Unaffiliated Fukushima Japan

**Keywords:** *Craseomys*, dental characteristics, taxonomy

## Abstract

We evaluated the molars in Anderson’s red-backed vole (*n* = 114) from the Kii Peninsula of Honshu, Japan. Two of the specimens are considered extremely old aged based on their dimensions and on the loss of alveolar capsules of M^2^, and a third one is also old based on its strongly worn left M^3^ and M_1_. Of the former two individuals, one showed an incipient closure of re-entrant angles at its basal end, as estimated from the difference between the occlusal patterns of the occlusal and basal surfaces of the left M_2_. The latter individual also showed a complete closure of the basal end in the left M^3^. These patterns differ from incipient roots observed in other vole taxa but were similar to a previous example of incipient roots in Anderson’s red-backed vole. Therefore, we suggest that molar roots in this species form at an extremely late age or by strong wear. Root formation in molars is considered an important diagnostic character, as *Eothenomys* molars lack roots, while *Craseomys* molars develop roots at a late age. However, this dental character may be particularly difficult to assess in voles under natural conditions. Considering previous phylogenetic findings based on molecular analyses, *Craseomys* is the most appropriate genus for Anderson’s and other Asiatic red-backed voles.

## ﻿Introduction

The taxonomic allocation of Anderson’s red-backed vole, *Eothenomysandersoni* (Thomas, 1905) (Rodentia, Cricetidae, Arvicolinae), is still a matter of discussion, as is that of Smith’s red-backed vole, *E.smithii* (Thomas, 1905) (Iwasa 2015a, 2015b). The distribution of Anderson’s red-backed vole is restricted to north-eastern and central Honshu and the Kii Peninsula of western Honshu, Japan (Iwasa 2015a). Previous studies of this vole taxon have disclosed intraspecific morphological and genetical variations (Aimi 1967, 1980; Miyao 1981; Tsuchiya 1981; Kitahara 1995; Kitahara and Harada 1996; Suzuki et al. 1999; Iwasa and Tsuchiya 2000; Iwasa and Suzuki 2002a, b, 2003). Various authors have varying opinions on its specific allocation: one species for all of the geographical populations (Kitahara 1995; Iwasa 2015a), two species for the north-eastern to central Honshu and the Kii Peninsula populations (Musser and Carleton 2005), or three species for the north-eastern Honshu, the central Honshu, and the Kii Peninsula populations (Imaizumi 1998). *Evotomys* Coues, 1874, *Craseomys* Miller, 1900, *Aschizomys* Miller, 1899, *Clethrionomys* Tilesius, 1850, *Phaulomys* Thomas, 1905, *Eothenomys* Miller, 1900, and *Myodes* Pallas, 1811 has been used for the species (Miller 1896, 1898; Thomas 1905; Anderson 1909; Tokuda 1941; Imaizumi 1960; Jameson 1961; Corbet 1978; Aimi 1980; Kawamura 1988; Corbet and Hill 1991; Musser and Carleton 1993, 2005; Kaneko and Murakami 1996; Luo et al. 2004; Shenbrot and Krasnov 2005; Suzuki et al. 2014; Iwasa 2015a, b; Kryštufek and Shenbrot 2022). Recent opinions have allocated Anderson’s red-backed vole to the genus *Craseomys* with other species having the *rufocanus* cytotype of the G-band patterns of chromosomes (Gamperl 1982; Modi and Gamperl 1989; Iwasa and Suzuki 2002b; Kohli et al. 2014; Tang et al. 2018; ASM (American Society of Mammalogists) Mammal Diversity Database, https://mammaldiversity.org/). In addition, according to Musser and Carleton (2005) and the ASM Mammal Diversity Database, a population of Anderson’s red-backed vole from the Kii Peninsula received specific rank, as *Myodes* (= *Clethrionomys*) *imaizumii* or *Craseomysimaizumii*, based on an assumed phylogenetically independent position (Iwasa et al. 1999; Suzuki et al. 1999).

At present, the vole has been assigned either to *Eothenomys*, *Myodes* (= *Clethrionomys*; see Kryštufek et al. 2020 concerning the availability of these two names for the genus of red-backed voles), or *Craseomys* (Kaneko and Murakami 1996; Musser and Carleton 2005; Iwasa 2015a, b; ASM Mammal Diversity Database; Tang et al. 2018; Kryštufek et al. 2020; Kryštufek and Shenbrot 2022). The different allocations are based on the possession or lack of root in its molars: *Eothenomys* (as a subgenus of *Microtus* in Miller 1896: 29, 44–47) has rootless molars and *Clethrionomys* (= *Evotomys* in Miller 1896: 29, 42–44) rooted ones, and *Craseomys* has molars developing roots late in life (Miller 1900: 87–91). Aimi (1980) studied allometric cranial measurements and molars of Anderson’s red-backed vole and referred it *Eothenomys* because of exclusively rootless molars in 416 individuals examined. Suzuki et al. (2014) also suggested that Anderson’s red-backed vole should be allocated as *Eothenomys* based on cytogenetic criteria. However, Jameson (1961) and Kitahara (1995) already had reported some teeth showing the beginnings of root formation: closed pulp cavities and incipient roots in one individual from a mountainous region of central Honshu (Jameson 1961: 599, 600); and signs of root closure, as in incipient roots, in the upper molars of one individual from the Kii Peninsula that had been kept in captivity (796 days old) (Kitahara 1995: 13). Consequently, Jameson (1961) allocated Anderson’s red-backed vole to *Clethrionomys*, whereas Kitahara (1995) classified this vole as an *Eothenomys*, considering the abnormal condition of growth without free occlusion due to excessive growth of the incisors. The generic allocation of Anderson’s red-backed vole has been discussed since its original description by Thomas (1905). The root condition of molars has been always an important argument for its generic status, even if it a bit ambiguous.

The purpose of the present study is to reconsider whether voles of this taxon have the potential to form roots in molars. We investigated signs of molar root formation, particularly in late-aged individuals in samples from the Kii Peninsula. On the basis of the current results, we re-evaluated the validity of the root condition of molars for generic determination in red-backed voles and tried to conclude the appropriate generic allocation of Anderson’s and other Asiatic red-backed voles.

## ﻿Materials and methods

Musser and Carleton (2005) distinguished two species within what has been known as Anderson’s red-backed vole, *Myodes* (= *Clethrionomys*) *andersoni* for populations from north-eastern and central Honshu, and *M.imaizumii* for a population from the Kii Peninsula of Honshu. However, these taxa are now considered to be conspecific because it is possible to obtain fertile offspring for several generations from their crosses (Kitahara 1995) and because molecular analyses show substantial differentiations within and among the populations (Iwasa and Suzuki 2002a, b, 2003; Iwasa 2008). Therefore, in this study, we consider these taxa conspecific in accordance with Iwasa (2015a). In total, 114 individuals of Anderson’s red-backed vole were collected using snap traps in the Wakayama Experimental Forest of Hokkaido University, Kozagawa, Wakayama Prefecture, Japan (33°39'N, 135°40'E), as shown in Appendix 1: Table A1. These individuals were collected in January to March, June, July, September, November, and December over 10 years (1986–1995) at the same sampling site, since collecting this species is difficult due to its low population density. For comparison, we also examined three individuals (HEG1-97, MAI-26, and MAI-347) of the grey red-backed vole, *M.rufocanus* (Sundevall, 1846) (Musser and Carleton 2005), from Hokkaido, Japan, that has rooted molars. These individuals are stored in the private collection in the laboratory of one of the authors.

**Figure 1.**
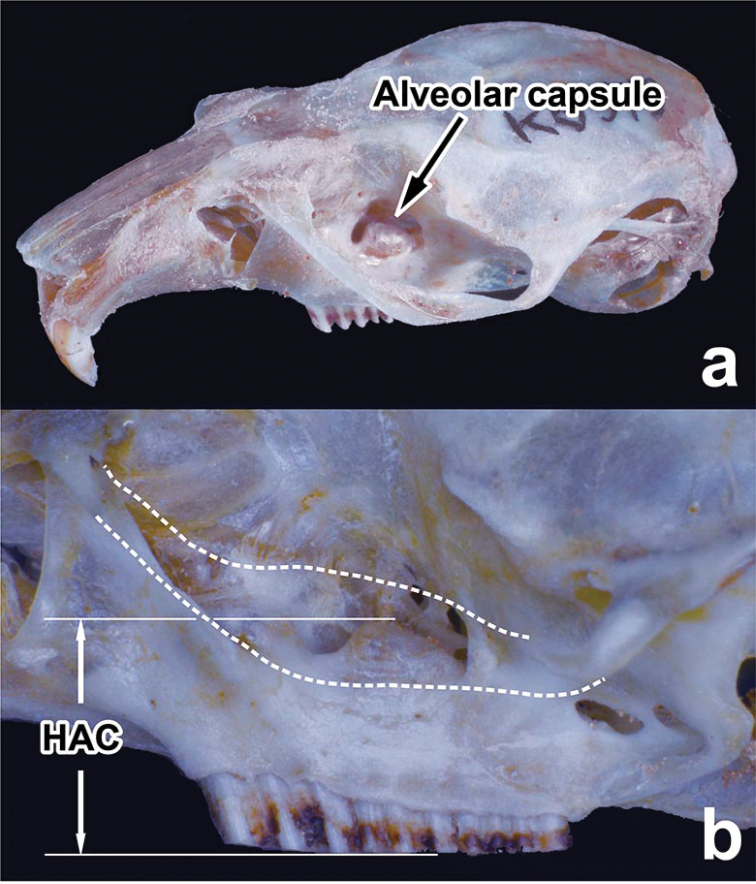
Position of the alveolar capsule **a** height from the occlusal surface of M^2^ to the upper edge of the alveolar capsule (HAC) at the left lateral view **b** dotted lines indicate outlines of the zygomatic arch, after its removal, to explain how the measurements were made.

Condylobasal length (CBL; the distance between the occipital condyle and the anterior point of the premaxillae) was measured to the nearest 0.1 mm using digital calipers. In addition, the height from the occlusal surface of the M^2^ to the upper edge of the alveolar capsules of M^2^ (HAC) was measured to the nearest 0.1 mm under a stereoscopic microscope using an objective micrometer (Kaneko 1988; Fig. 1; Appendix 1: Table A1). Moreover, from some skulls (HEG1-97, MAI-26, MAI-347, K6059, K7088, K7344, and K7367), we removed the molars and checked the enamel patterns at the occlusal and basal ends to detect a possible closure of the basal end, which would infer a root formation.

We defined adults as individuals that had reached sexual maturation (Appendix 1: Table A1) by correspondence to any of the following genital conditions: appearance of the papilla mammae, opening of the pubic symphysis, pregnancy, and the presence of placental scars in females, and the presence of the ductus epididymis at the cauda epididymis and testes larger than 7.5 mm in males. For references to the aging variation, external dimensions were measured and were described in Appendix 1: Table A1 as follows: body weight (BW), head and body length (HB), tail length (T), and hind foot length sine-unguis (HF).

## ﻿Results

**Figure 2.**
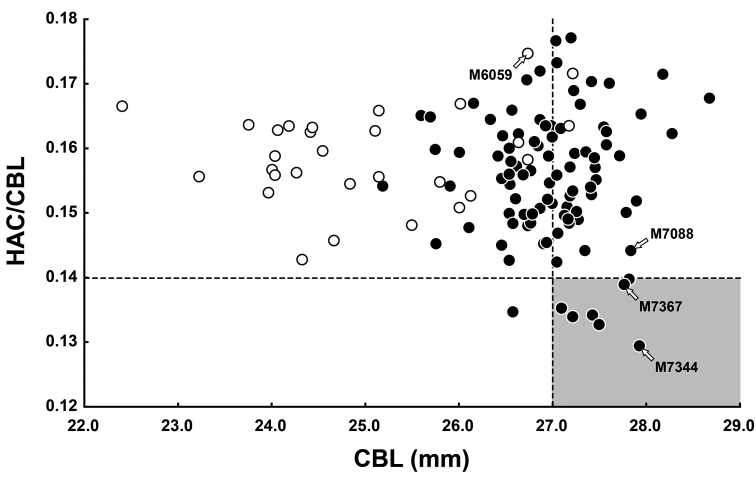
Scatter plots of a relationship between CBL and HAC/CBL. We recognized individuals showing quite lower HAC by the following definitions as old aged (greyish zone, see text): HAC/CBL ≤ 0.14 and CBL ≥ 27.0 mm. White and black circles indicate immature and mature individuals, respectively (Appendix 1: Table A1). Arrows indicate individuals showing incipient roots (see text and Appendix 1: Table A1).

The studied individuals (*n* = 114) were determined as immature (*n* = 30) or mature (*n* = 84) ones based on their genital conditions (Appendix 1: Table A1). According to Kaneko (1990), the alveolar capsules of M^2^ disappear in red-backed voles during root formation. Thus, we primarily analysed the relationship between HAC/CBL and CBL, displayed in a scatterplot (Fig. 2). This relationship indicated that sexually immature individuals showed a HAC/CBL > 0.14. In addition, we referred individuals with a CBL ≥ 27.0 mm as mature because most immature individuals showed a CBL ≤ 27.0 mm (Fig. 2). On the basis of these discriminations, individuals with both HAC/CBL < 0.14 and CBL ≥ 27.0 mm were considered to be of extremely old age under natural condition, which probably correlates with an age of more than one year (Kitahara 1995). Namely, seven individuals included in the cluster with both HAC/CBL < 0.14 and CBL ≥ 27.0 mm (greyish zone of Fig. 2; Appendix 1: Table A1) were studied for molar characteristics.

**Figure 3.**
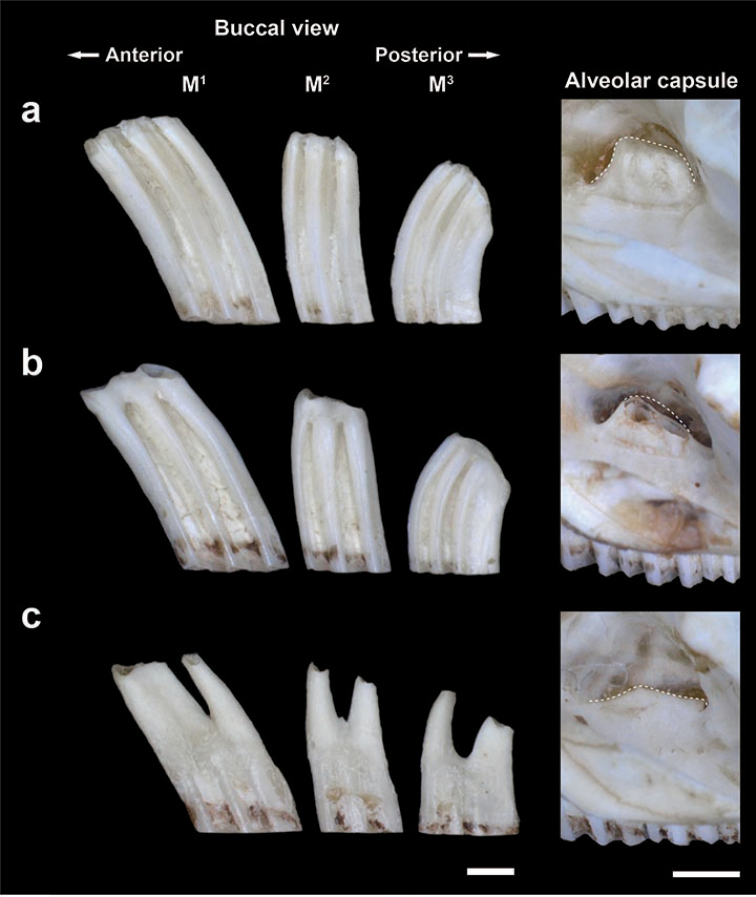
Typical buccal views of the left upper molars and alveolar capsule conditions of the grey red-backed vole showing a non-rooted type **a** MAI-26, an incipient rooted type (**b** HEG1-97 inversed image of a right capsule) and a completely rooted type **c** MAI-347. Dotted lines indicate outlines of alveolar capsules (partially broken in HEG1-97). Scale bars: 1 mm.

As a control group for the molar root condition, we documented three adult individuals of the grey red-backed vole with rooted molars as in Fig. 3 (Kaneko 1990; Nakata 2015). From these, the individuals MAI-26 and HEG1-97 were considered to be relatively younger, because one showed a higher alveolar capsule and no signs of root formation and the other showed a moderately higher alveolar capsule and root formation, respectively, whereas the individual MAI-347 showed a completely formed root and the alveolar capsule was lost.

**Figure 4.**
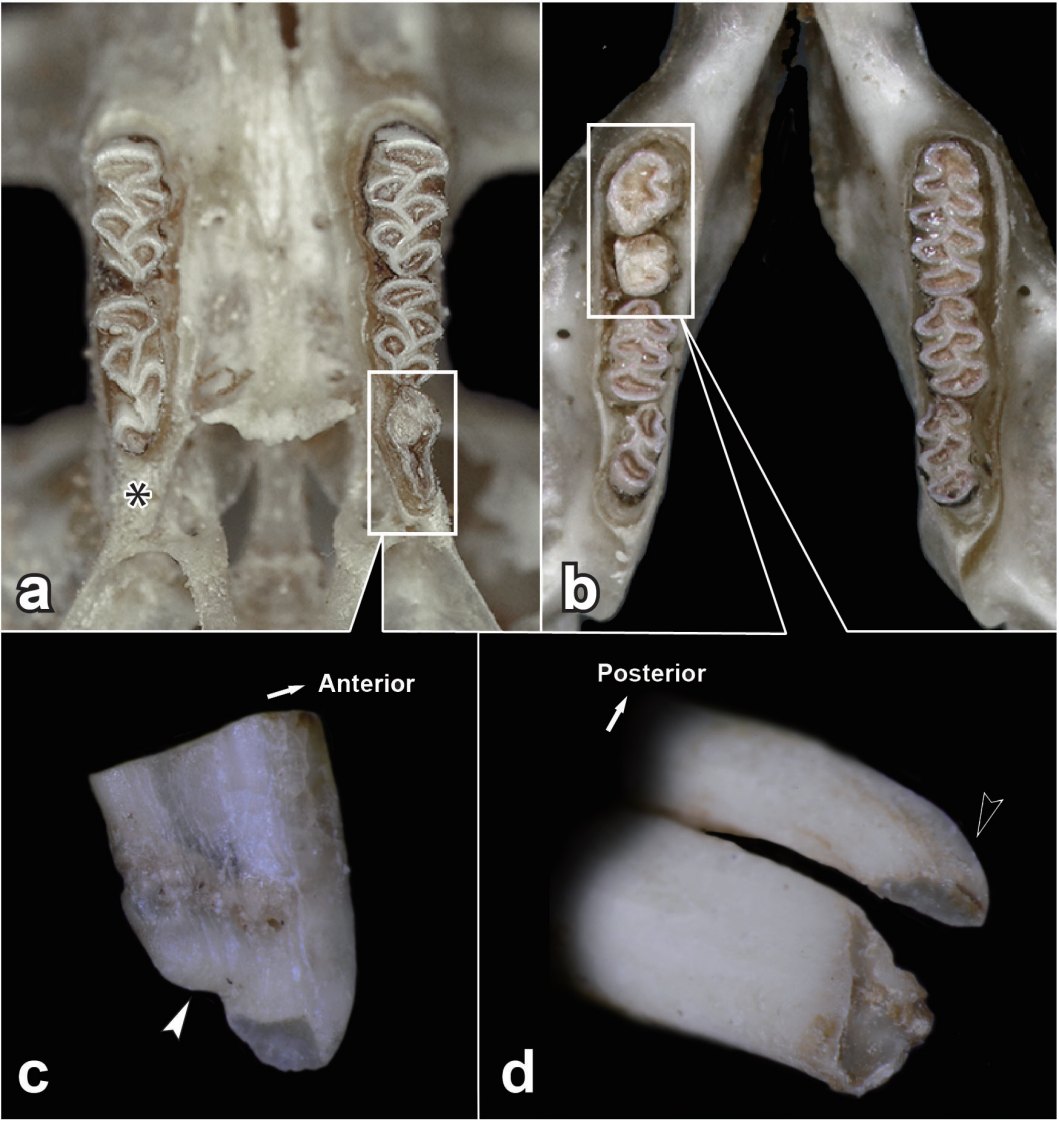
Occlusal views of the upper **a** and lower **b** tooth rows, buccal view of the left M^3^**c** and antero-buccal view of the left M_1_**d** of individual K7088. Asterisk indicates an abnormal lack of the right upper third molar. Black and white arrowheads indicate a bent basal end and a complete closure of re-entrant angles at the basal end, respectively.

**Figure 5.**
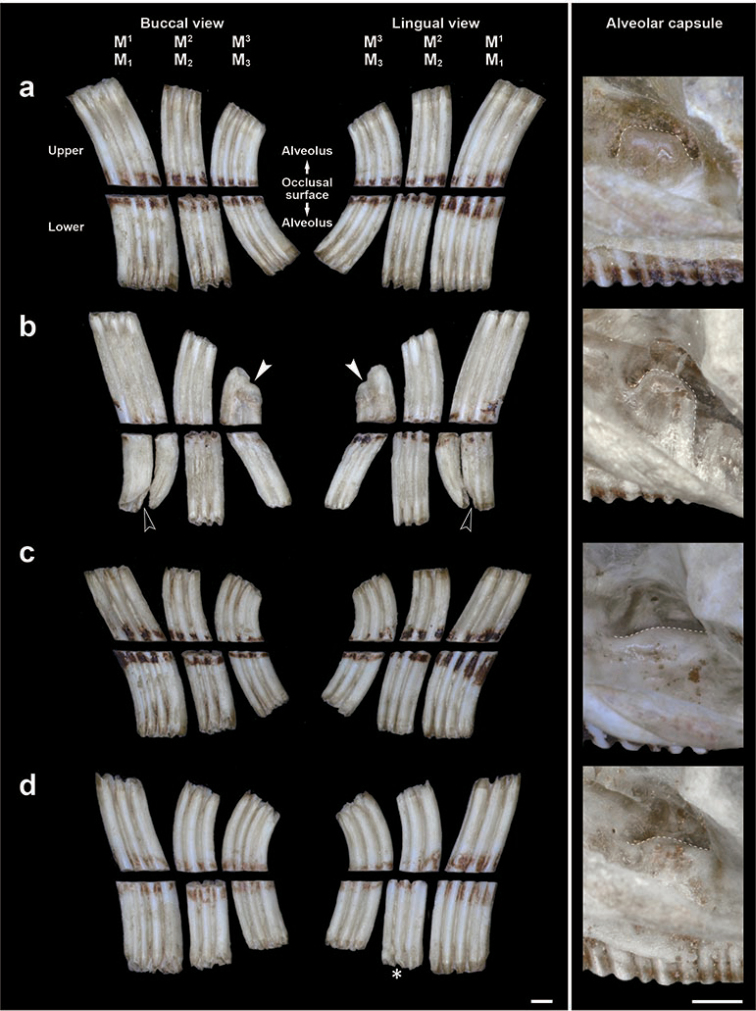
Typical buccal and lingual views of the left upper and lower molars and alveolar capsule conditions of four individuals of Anderson’s red-backed vole **a** K6059 **b** K7088 **c** K7344 **d** K7367. Arrowheads indicate a root-like strong crevice caused by a crack. Basal ends indicated by white arrowheads and asterisks are considered to be complete closures of re-entrant angles as a root at the basal end and an incipient closure of re-entrant angles, leading to incipient root formation, respectively. Black arrowheads indicate abnormal cracks. Dotted lines indicate outlines of alveolar capsules. Scale bars: 1 mm.

In the seven extremely old-aged individuals of Anderson’s red-backed vole, we checked the condition of the basal ends of the molars. Of the seven individuals, two (K7344 and K7367) showed a loss of the alveolar capsules of M^2^. In addition, one individual (K7088) showed an extremely worn molar crown (Fig. 4a, b). Both buccal and lingual views of the left upper and lower molars and the alveolar capsule conditions of these individuals and of one with an apparent high alveolar capsule (K6059) are shown in Fig. 5. In K6059, all the basal ends of the tooth crown were open, and grooves occurred between the occlusal surfaces and the basal ends, in combination with a high alveolar capsule. In contrast, of the two individuals which lost their alveolar capsule, K7367 showed that the basal end tapered off (indicated by asterisks in Fig. 5) in M_2_. In addition, K7088 displayed that the basal end of M^3^ showed a complete closure (indicated by white arrowheads in Figs 4, 5), irrespective of having a higher alveolar capsule of M^2^ (Appendix 1: Table A1). Moreover, the occlusal surface of M_1_ was cracked and split into two parts, and the basal end of the posterior part of M_1_ was bent in the anterior direction and tapered off (indicated by black arrowheads in Figs 4, 5). Interestingly, the individual K7088 demonstrated that, as a rare example, the right M^3^ was lacking and the right M^2^ was elongated to the posterior part, and the left side of M_1_ was extremely worn as compared with the right M_1_ (Figs 4, 5).

**Figure 6.**
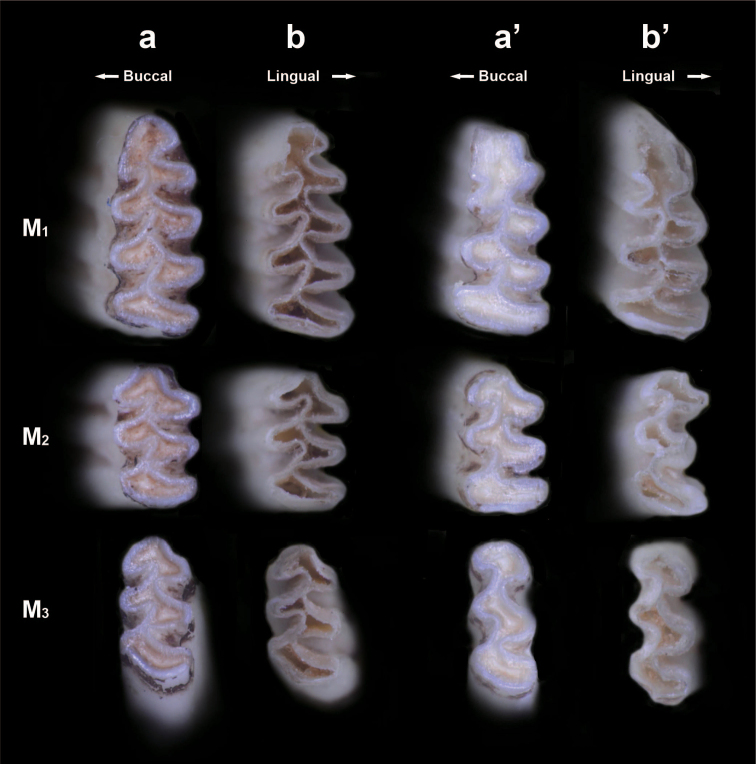
Typical views of occlusal surfaces **a, a**’ and basal ends **b, b**’ of the left lower molars of two typical individuals **a, b** K6059 with alveolar capsules **a’, b**’ K7367 without them of Anderson’s red-backed voles. Inversed images indicate whether basal end views **b, b**’ correspond to the enamel patterns of occlusal surfaces **a, a**’.

**Figure 7.**
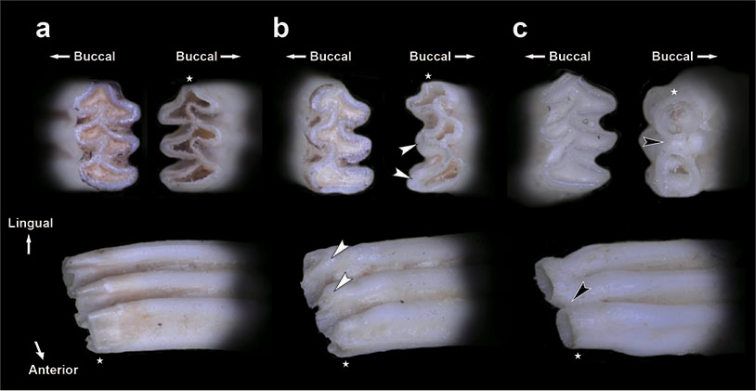
Typical views of occlusal surfaces (above left) and basal ends (above right), and antero-lingual views (below each) of M_2_ of three typical individuals **a** K6059 with alveolar capsules **b** K7367 without them of Anderson’s red-backed voles and the grey red-backed vole **c** HEG1-97 with the middle height of the alveolar capsule showing an incipient root. Black and white arrowheads indicate that the basal end was completely closed and that the basal ends were dully tapered off, showing incipient closures of re-entrant angles, respectively. Stars indicate correspondences of anterior points.

Furthermore, we observed the enamel patterns of occlusal surfaces and the basal ends of the molars in detail with higher magnification, shown in Figs 6, 7. The enamel patterns of the basal ends corresponded completely to the enamel patterns of occlusal surfaces in individuals with apparent alveolar capsules and/or no sign of incipient closure of re-entrant angles at the basal ends, as in K6059 (Fig. 6), for example. On the other hand, in K7367, which lacked alveolar capsules, most molars showed the same situation as in K6059, but the basal ends of the left M_2_ were dully tapered off as incipient closures of re-entrant angles (Figs 6, 7). Therefore, the enamel pattern of the occlusal surface of M_2_ was apparently different from that of the basal ends of M_2_ (Figs 6, 7). An incipient root formation of M_2_ (HEG1-97) of the grey red-backed vole also showed that the enamel shape of the basal ends was completely different from that of its occlusal surface (Fig. 7).

## ﻿Discussion

Thomas (1905) in his original description, allocated Anderson’s red-backed vole to the genus *Evotomys*, which had been erected by Miller (1896), until the priority of *Clethrionomys* had been discovered by Palmer (1928), and later to *Eothenomys*, then a subgenus of *Microtus*, because roots in molars were considered being absent. Following Miller (1896), Jameson (1961) classified this vole as *Clethrionomys*. Also, Corbet (1978) and Corbet and Hill (1991) designated it as *Clethrionomys*, following Miller (1896) and Jameson (1961). In addition, Musser and Carleton (2005) expanded the definition of *Myodes* (= *Clethrionomys*) to include species with and without rooted molars, allocating it to the genus *Myodes* based on molecular studies. On the other hand, some Japanese taxonomists have allocated Anderson’s red-backed vole to the genus *Eothenomys* because it was believed that this taxon had rootless molars (Aimi 1980; Kitahara 1995; Kaneko and Murakami 1996; Iwasa 2015a, b), but the ASM Mammal Diversity Database recently assigned the Asian vole species *rufocanus*, *rex*, *andersoni*, *smithii*, *regulus*, and *shanseius* to the genus *Craseomys*, according to Abramson and Lissovsky (2012) and Kohli et al. (2014). This opinion is in good accordance with the karyological findings that red-backed voles are divided into two lineages, the *glareolus* cytotype group in the Holarctic and Nearctic realms and the *rufocanus* cytotype group in the Palaearctic realm, based on the G-band patterns. At least all of above members of Asian red-backed voles show the *rufocanus* cytotype as a monophyly (Gamperl 1982; Modi and Gamperl 1989; Jiang and Ma 1991; Iwasa and Suzuki 2002b; Tang et al. 2018).

Jameson (1961) and Kitahara (1995) had previously studied the molar root formation of Anderson’s red-backed vole, but little attention has been paid elsewhere. Jameson (1961: 599–600) mentioned the presence of incipient roots in one of the nine individuals of the vole from the central mountain region (Yatsugatake Mountains) of Honshu and allocated it as *Clethrionomys*. However, for the nine individuals likely investigated by Jameson (1961), one individual (USNM399102, preserved in the Smithsonian National Museum of Natural History) and four individuals (2565z, 2572z, 2777z, and 2778z, preserved in the Museum of Wildlife and Fish Biology of the University of California, Davis) were investigated by one of the present authors (Y. Kaneko) and the curator of the MWFB of UC Davis (A. Engilis Jr.) for the root conditions of molars. Contrary to Jameson’s (1961) observation, it was confirmed that these individuals do not carry an incipient root condition. To date, unfortunately, another individual (M-184568, preserved in the American Museum of Natural History) has not been investigated, and the three other individuals are missing.

In the M_1_ of an individual of *Ondatrazibethicus* (Linnaeus, 1766) in which the roots are not yet expressed, the re-entrant angles in the alveolar basal part are completely closed (Borodin 2009: fig. 11-2-d). Such a closed alveolar basal part has been confirmed in other voles (Gromov and Erbajeva 1995; Koenigswald and Kolfschoten 1996; Borodin 2009). In all of the molar samples showing these incipient root conditions, the enamel patterns of the occlusal surfaces do not commonly repeat those of the basal ends and both patterns do not correspond. Therefore, we consider that such discordance between the enamel patterns of the occlusal surface and the basal ends (Figs 4, 7) is a sign of an incipient closure of the re-entrant angles at the basal ends, reaching an incipient root formation. On the other hand, Kitahara (1995) regarded the signs for root-closure in one individual kept in captive conditions (796 days old) collected from the Kii Peninsula as an abnormal condition of growth without free occlusion due to excessive growth of the incisors, therefore allocating the vole as *Eothenomys*. According to the photographs of these root-closure molars (Kitahara 1995: 13B), the basal ends of M_1_, M_2_, and M_3_ were apparently tapered off from the occlusal surfaces, and the grooves were still clearly formed from the occlusal surface to the basal ends, particularly in M_3_. In addition, the middle portion of M_2_ was abnormally bulged, and such bulging has not been confirmed in voles. These characteristics were apparently different from those of the so-called incipient roots and roots of molars in other arvicolines, such as the grey red-backed vole (Fig. 3b, c) and *O.zibethicus* (Borodin 2009). However, the features of the basal end of M_1_ of Kitahara (1995) are similar to those of M^3^ of K7088 as an incipient root, as caused by the abnormally strong wearing of M^3^ in K7088 (Fig. 4). It is considered that the characteristics of the basal end of K7088 might be caused by an abnormal occlusion due to a lack of right M^3^ (Fig. 4). In addition, those of the basal end of M_1_ of Kitahara (1995) are also similar to those of M_2_ of K7367 as an incipient closure of re-entrant angles (Figs 6, 7). The current observation does not correspond to previous findings by Gromov and Erbajeva (1995) and Borodin (2009), as to the typical incipient root status. However, we suggest that the current characteristics, the basal ends tapered off as in Kitahara (1995), the discordance of the enamel patterns between the occlusal surface and the basal ends, and the loss of alveolar capsule of M_2_ (Figs 4, 5, 7) would be regarded as early stages reaching into molar root formation. Accordingly, the current characteristics of the basal ends of molars mentioned above suggest that molar root formation potentially appears at an extremely old-aged stage of life or by strong wearing in Anderson’s red-baked vole, corresponding to the characteristic of *Craseomys*, with roots of molars that develop late in life (Miller 1900) rather than that of *Myodes*.

Considering the cytotype phylogenetic relationships and the dental characteristics of the root formation period in molars, ASM Mammal Diversity Database’s allocation of Anderson’s red-backed vole in *Craseomys* is acceptable. However, Anderson’s red-backed vole has similarities with genus *Eothenomys* for by two reasons. First, incipient roots were present only in individuals of the Kii Peninsula, including one starving individual reared by Kitahara (1995), and it is unclear whether root formation is present in Anderson’s red-backed voles collected from other localities of central and northern Honshu. Second, morphological and phylogenetic findings disclosed the close relationship between Anderson’s red-backed vole and Smith’s red-backed vole (Kaneko et al. 1992; Kimura et al. 1994, 1999; Suzuki et al. 1999; Iwasa and Tsuchiya 2000; Iwasa and Suzuki 2002a, b, 2003; Fujimoto and Iwasa 2010; Iwasa 2015b), and rooted molars have never been reported in Smith’s red-backed vole to date (Imaizumi 1949, 1960; Tanaka 1971; Aimi 1980).

In our study, two individuals (K7088 and K7367) showed incipient root conditions and the incipient closure of the re-entrant angles in the molars among 114 individuals of Anderson’s red-backed vole from the Kii Peninsula (Figs 4, 5, 7). These two were found among 114 individuals collected in all months except April, May, and October, suggesting that this molar condition is not specific but a normal phenomenon in the field. Our vole sampling was carried out in just a few days per year, as sampling of the vole is very difficult due to its low density and its specific habitat in rocky terrain, as compared to mice of the genus *Apodemus* which are dominant in the Japanese Islands (Iwasa 2008, 2015a). Considering such limited sampling of the voles, the determination of the period of molar root formation is difficult using vole samples caught in natural conditions, whose true ages are unknown. Particularly, such difficulty would be expected in red-backed voles showing molar root formation at late age stages, as in the present results, because longevity in these animals in natural conditions is usually ecological rather than physiological. The difficulty of confirming molar root formation has probably caused the confusion in the genus allocation, and the dental feature may not be realistic for the generic classification of red-backed voles, particularly Anderson’s and Smith’s red-backed voles, which are apparently closely related. Therefore, we suggest that *Craseomys* is the most appropriate genus for Anderson’s red-backed vole and other Asiatic red-backed voles, including Smith’s red-backed vole. Our suggestion agrees with Kryštufek and Shenbrot (2022) and the ASM Mammal Diversity Database, and it considers the karyological and molecular phylogenetic relationships (Modi and Gamperl 1989; Iwasa and Suzuki 2002b; Tang et al. 2018).

## ﻿Appendix 1

**Table A1.** Anderson’s red-backed vole individuals examined in this study.

No.	Sex	BW (g)	TL* (mm)	HBL (mm)	HFLsu (mm)	Testis length (mm)	C. e.*	P. m.*	P. sym.*	Pg.*	P. s.*	CBL (mm)	HAC (mm)	Mat.*		No.	Sex	BW (g)	TL* (mm)	HBL (mm)	HFLsu (mm)	Testis length (mm)	C. e.*	P. m.*	P. sym.*	Pg.*	P. s.*	CBL (mm)	HAC (mm)	Mat.*
K5619	m	26.5	55.7	104.5	19.7	3.5	–					25.1	3.91	Im		K6795	m	33.9	61.2	111.7	19.5	8.5	+					25.9	3.99	M
K5620	f	31.5	59.0	117.4	18.9			+	–			nd	4.44	M		K6796	f	38.1	71.5	112.5	19.2			+	nd			26.8	3.97	M
K5646	f	24.2	61.5	105.7	19.6			–	+			24.5	3.92	Im		K6797	m	25.3	61.0	98.9	19.2	7.0	–					24.7	3.60	Im
K5647	f	37.5	71.9	112.3	19.3			+	nd			26.5	4.10	M		K6804	f	31.2	62.5	108.8	19.0			–	+			26.6	4.29	Im
K5648	m	37.5	62.5	118.2	20.2	5.6	–					27.2	4.67	Im		K6805	f	35.3	72.3	104.2	19.5			+	nd			27.5	3.65	M
K6034	m	42.0	64.2	123.1	20.8	9.2	+					27.3	3.94	M		K6806	m	27.6	59.8	101.9	19.5	6.0	-					25.5	3.78	Im
K6035	f	36.5	66.9	112.8	20.4			+	–			27.0	4.17	M		K6807	m	35.6	66.3	110.7	20.2	7.6	+					26.0	4.15	M
K6036	m	38.0	62.3	122.2	19.5	8.4	+					27.3	4.07	M		K6808	m	38.7	62.9	120.6	19.3	9.0	+					27.2	4.04	M
K6037	m	40.4	62.7	118.2	20.5	9.6	+					27.7	4.40	M		K6810	f	37.0	70.0	119.5	21.2			–	–			27.4	4.22	M
K6038	f	37.0	71.7	116.0	20.4			+	–	+		26.5	3.98	M		K6811	m	31.2	63.7	110.2	19.9	8.6	+					26.5	4.25	M
K6039	m	38.9	65.6	123.5	20.1	8.5	+					26.6	4.41	M		K6812	m	32.1	57.2	112.5	20.2	6.2	–					26.7	4.23	Im
K6040	m	44.0	63.3	122.4	19.5	8.9	+					27.5	4.31	M		K6813	m	34.5	65.1	114.2	20.4	7.7	+					26.8	4.31	M
K6041	f	38.2	67.6	113.8	19.6			+	–			27.2	4.10	M		K6814	f	44.9	68.8	117.8	20.4			+	–	+		27.9	4.62	M
K6042	f	35.1	73.1	117.2	19.6			+	–	+		27.4	4.19	M		K6815	f	34.6	63.2	114.2	19.8			+	–		+	27.0	4.78	M
K6043	f	35.5	56.8	112.9	20.0			+	–			26.1	3.86	M		K6816	f	31.9	63.7	106.2	19.4			+	–			26.2	4.37	M
K6044	m	44.6	62.5	122.2	21.2	8.6	+					27.2	3.65	M		K6817	m	41.7	67.9	113.4	19.8	7.8	+					27.3	4.10	M
K6052	m	41.0	66.5	122.9	20.1	8.3	+					27.0	4.41	M		K6818	m	33.3	67.7	109.6	20.2	8.5	+					nd	nd	M
K6053	f	45.4	65.2	120.1	20.0			+	–	+		27.0	4.22	M		K7085	m	22.2	54.9	101.1	19.2	6.7	–					24.4	3.97	Im
K6054	m	37.0	58.8	117.8	20.0	9.2	+					27.0	3.85	M		K7086	m	19.5	54.0	90.1	19.5	5.1	–					23.8	3.89	Im
K6055	f	45.4	65.3	118.0	19.5			+	nd			27.1	3.97	M		K7087	m	42.2	65.2	119.9	21.5	9.6	+					27.9	4.24	M
K6056	f	34.3	60.5	113.5	20.4			+	+	+		26.6	4.19	M		K7088	m	43.5	62.2	123.9	20.5	9.5	+					27.8	4.02	M
K6057	f	40.8	57.8	118.8	19.1			+	–			27.0	4.09	M		K7089	m	24.8	55.8	99.7	20.2	5.9	–					24.4	3.99	Im
K6058	m	39.0	64.4	117.2	19.8	8.8	+					26.9	3.91	M		K7090	f	42.9	64.0	119.3	19.0			+	nd	+	+	nd	4.16	M
K6059	m	31.5	61.3	111.2	19.8	6.0	–					26.7	4.67	Im		K7100	f	23.9	43.0+	96.3	19.6			+	+			24.0	3.75	Im
K6070	m	42.5	nd	121.9	20.2	10.0	+					27.8	3.89	M		K7101	f	28.6	59.7	112.0	20.2			+	–			25.6	4.23	M
K6071	f	41.0	62.3	117.7	19.5			+	–			27.2	4.27	M		K7102	m	17.9	48.8	85.9	19.2	4.2	–					22.4	3.73	Im
K6072	m	42.8	50.2	123.4	19.2	9.2	+					27.4	3.68	M		K7103	m	26.9	58.8	101.2	19.2	5.2	–					24.2	3.95	Im
K6073	f	36.5	64.9	112.5	20.2			+	–	+		26.9	4.42	M		K7104	f	42.3	68.2	114.0	20.0			+	–	+		27.1	4.06	M
K6074	m	40.9	64.5	122.2	19.9	8.9	+					27.1	3.67	M		K7105	m	28.1	58.8	94.7	19.4	6.1	–					24.0	3.82	Im
K6075	f	31.0	71.9	110.2	20.3			+	+			nd	4.07	M		K7106	m	28.3	59.5	102.2	19.7	5.0	–					24.3	3.79	Im
K6118	m	26.2	61.9	100.9	20.1	3.0	–					25.1	4.17	Im		K7113	m	41.8	58.3+	120.0	19.5	8.7	+					27.1	4.42	M
K6119	f	31.5	67.8	116.3	20.3			+	–			26.7	3.96	M		K7114	f	42.7	65.2	121.2	20.6			+	–		+	27.2	4.60	M
K6442	m	34.4	59.9	114.8	20.8	9.6	+					26.3	4.33	M		K7115	m	43.1	64.3	118.2	19.4	8.6	+					27.2	4.05	M
K6537	m	25.9	57.0	96.2	20.2	6.1	–					nd	3.78	Im		K7116	m	40.0	68.1	120.0	19.9	8.4	+					27.2	4.18	M
K6538	f	31.1	59.0	111.1	20.0			–	+			25.8	3.99	Im		K7117	f	44.6	70.4	123.8	19.9			+	–			28.7	4.81	M
K6539	f	46.9	66.5	118.2	19.3			+	–	+	+	27.2	4.34	M		K7118	f	49.1	67.2	121.6	19.2			+	–			27.6	4.43	M
K6540	f	31.8	62.2	111.7	20.0			+	–			25.7	4.12	M		K7119	f	38.6	69.5	111.2	20.7			+	–	+		26.6	4.32	M
K6541	f	18.6	53.7	92.1	19.2			–	+			23.2	3.62	Im		K7120	m	39.3	72.5	117.2	20.4	7.9	+					26.9	4.62	M
K6542	f	25.5	61.5	100.4	20.5			–	+			24.1	3.92	Im		K7121	f	39.0	67.7	116.7	19.8			+	–			26.7	4.56	M
K6543	f	28.1	59.4	107.1	20.8			–	+			24.8	3.84	Im		K7122	m	45.3	67.6	125.2	20.4	9.2	+					28.2	4.83	M
K6559	f	46.4	69.0	120.3	20.0			+	–			27.6	4.70	M		K7123	f	43.2	63.3	119.6	19.7			+	–	+		26.9	4.10	M
K6560	f	49.1	68.5	125.5	20.8			+	–			28.3	4.59	M		K7124	f	37.7	61.0	115.6	19.8			+	–		+	26.4	4.20	M
K6561	f	40.1	67.6	119.5	20.4			+	–	+		26.6	3.94	M		K7125	m	42.7	70.0	122.1	20.6	9.2	+					27.4	4.36	M
K6562	m	38.5	69.2	116.5	20.0	8.4	+					26.8	4.19	M		K7340	f	38.9	63.8	116.4	19.4			+	–	+		26.6	4.20	M
K6563	m	25.5	63.5	98.0	19.6	6.2	–					24.3	3.47	Im		K7341	m	43.7	64.6	121.3	21.0	10.0	+					27.5	4.50	M
K6564	m	34.0	58.2	109.8	20.9	8.5	+					26.5	4.29	M		K7342	m	33.5	60.0	112.6	20.9	8.6	+					26.5	4.14	M
K6565	m	33.5	57.2	111.8	19.3	8.5	+					25.2	3.88	M		K7343	m	35.1	58.2	119.2	19.2	9.5	+					26.5	3.84	M
K6575	f	40.2	74.2	111.5	20.3			+	nd			27.2	4.15	M		K7344	f	48.2	63.0+	125.2	20.2			+	–	+		27.9	3.62	M
K6576	f	25.9	60.3	97.1	20.2			–	+			24.0	3.76	Im		K7345	f	45.7	63.8	118.2	20.5			+	–	+		26.7	4.00	M
K6577	f	22.7	51.9	96.5	19.2			–	+			24.0	3.67	Im		K7346	m	39.8	59.6	117.7	20.0	10.1	+					27.2	4.82	M
K6781	m	31.0	70.0	115.2	20.5	8.5	+					26.5	4.11	M		K7347	f	44.2	66.1	121.6	20.0			+	–			27.8	4.17	M
K6782	f	34.3	62.0	116.8	19.8			nd	–		+	26.5	3.79	M		K7348	m	32.7	65.1	112.6	20.2	7.2	–					26.0	3.92	Im
K6783	f	36.6	73.0	125.6	20.2			+	–			27.5	4.26	M		K7354	m	38.3	59.0	115.6	19.2	10.1	+					26.8	4.32	M
K6784	m	36.6	67.9	117.5	20.0	7.2	+					27.4	4.67	M		K7355	f	36.8	67.5	115.3	20.2			+	–	+		26.8	4.02	M
K6785	f	31.0	64.3	113.2	20.0			–	+			26.1	3.99	Im		K7367	f	40.4	71.0	128.5	19.8			+	–			27.8	3.85	M
K6786	m	32.8	65.3	111.2	19.8	8.0	+					26.7	4.16	M		K7368	f	42.1	70.6	121.0	20.1			+	–		+	27.4	4.35	M
K6787	f	32.4	66.2	113.6	19.4			+	–			26.6	4.05	M		K7369	f	34.3	64.3	112.9	19.2			+	–			25.8	3.74	M
K6788	f	28.9	63.7	104.5	19.3			–	+			26.0	4.34	Im		K7370	m	28.0	61.6	105.2	20.2	7.0	–					25.1	4.09	Im
K6789	f	34.7	63.2	118.2	19.4			nd	–			27.0	4.37	M		K7371	m	44.3	68.2	125.3	20.3	10.5	+					27.6	4.48	M
K6790	f	34.8	64.5	115.0	19.0			–	nd			26.6	3.58	M		K7372	f	32.9	65.9	113.5	20.2			+	–			25.7	4.24	M
K6791	m	38.8	66.2	117.1	20.5	8.0	–					27.2	4.44	Im		K7373	f	39.7	62.5	119.2	19.2			+	–			26.9	4.40	M
K6792	m	41.0	63.2	118.1	19.9	8.8	+					27.3	4.56	M		K7374	m	40.4	63.2	122.3	20.0	9.4	+					26.9	3.92	M
K6793	m	33.8	61.4	114.8	20.6	8.8	+					26.9	4.05	M		K7375	m	40.4	55.0+	122.0	20.2	9.6	+					27.0	4.69	M
K6794	f	34.2	69.5	109.9	20.5			+	–			27.0	4.28	M																

*+ in TL, tail partially lost; C. e., cauda epididymidis (+: ductus epididymis appear, -: ductus epididymis disappear); P. m., papilla mammae (+: appear, -: disappear); P. sym., pubic symphysis (+: close, –: open); Pg., Pregnance; P. s., placental scars; Mat., sexually maturation (Im: immatured, M: matured). nd, no data.
